# Proof of Concept of Culturomics Use of Time of Care

**DOI:** 10.3389/fcimb.2020.524769

**Published:** 2020-11-23

**Authors:** Sabrina Naud, Saber Khelaifia, Maxime Descartes Mbogning Fonkou, Niokhor Dione, Jean-Christophe Lagier, Didier Raoult

**Affiliations:** ^1^ Aix Marseille Univ, IRD, AP-HM, France, MEPHI, Marseille, France; ^2^ Institut Hospitalo-Universitaire Méditerranée Infection, Marseille, France

**Keywords:** culturomics, gut microbiota, fastidious bacteria, anti-PD1 monoclonal antibodies, rapid culture

## Abstract

Culturomics, a high throughput culture method with rapid identification of the colonies by Matrix Assisted Laser Desorption Ionization/Time Of Flight Mass Spectrometry (MALDI-TOF MS), has demonstrated its contribution to the exploration of the gut microbiota over the past 10 years. However, the cost, work time and workload, considerably limit its use on a large scale or emergency context. Here, by testing two different stool samples, including a stool sample from a patient requiring rapid immunotherapy treatment, we tested a new fast culturomic protocol using two pre-incubation media, blood culture bottle and YCFA modified medium. Both media were supplemented with 2 ml of rumen fluid filtered at 0.2 μm and 2 ml of defibrinated and sterile sheep blood. Unlike the standard culturomics, subculturing of blood culture bottle were performed at reduced incubation time (3 h, 6 h, 9 h, 24 h) and at a longer incubation time (3 days, 7 days, and 10 days) at 37°C. By testing 5,200 colonies per MALDI-TOF MS and obtaining a comparable number of cultured bacterial species (131 to 143) in a stool sample, this new protocol reduced the number of colonies tested by 57%, working time by 78.6% and cost by 72.2%. In addition, we highlighted that the proportion of strict anaerobic species has increased by 24%, known to be the preferential targets for biotherapy, including *Faecalibacterium prausnitzii*, *Akkermansia muciniphila, Christensenella minuta*, and *Phascolarctobacterium faecium.* Finally, this work showed that some bacterial species grew earlier but disappeared with prolonged incubation times.

## Introduction

The culturomics of the bacterial species of the digestive tract probably represents an indispensable step toward biotherapy in the coming years for colitis, especially *Clostridioides difficile* colitis (Amrane et al., 2019), but also in the context of obesity (Everard et al., 2013), malnutrition (Tidjani Alou et al., 2017) and cancer immunotherapy (Lagier et al., 2019). In this context, the problematic aspect of culturomics is that, in its current state, it is extremely restrictive in terms of time, equipment and culture medium (Lagier et al., 2012). Thus, on average, in recent years, to have a clear vision of the cultivable microbial flora, we have to test 18 different culture conditions and test 10,000 spots by MALDI-TOF MS to obtain 100 different bacterial species (Lagier et al., 2015a). Moreover, it is becoming increasingly clear that strict or facultative anaerobic bacterial species are the preferred biotherapy targets (Duncan et al., 2002; Daillère et al., 2016; Dao et al., 2016; Tanoue et al., 2019). We were able to have a case for proof of concept in a patient (who is our colleague) in whom we wanted to find the bacterial species positively associated with the immunotherapy support. This allowed us to compare a method to quickly determine, before treatment was initiated, whether he had the bacterial species associated with a response to his treatment with antiPD-1 monoclonal antibody at the time of treatment (Lagier et al., 2015b; Routy et al., 2018).

## Materials and Methods

### Patients and Samples

During this work, two stools were grown in the culturomics laboratory of the “Institut Hospitalo-Universitaire Méditerranée Infection” in Marseille, France to explore the intestinal microbiota of these individuals. In the urgent need to obtain the exploration of the intestinal microbiota of a patient requiring rapid treatment with anti-PD1 (Routy et al., 2018), we analyzed his stool sample using a new fast culturomics protocol (see methods, Section culture). We cultivated a first stool sample from a 59-year-old Caucasian male patient. The second sample was collected from a 29-year-old healthy Caucasian woman. The stool was placed in a between-open stool culture pot and placed in a Zip bag (Oxoid, Dardilly, France) containing an anaerobic GasPak (Becton Dickinson, Le Pont de Claix, France) to undermine the anaerobic atmosphere. The stools were then introduced directly into an anaerobic chamber (Don Whitley Scientific, Bingley, UK) with limited oxygen exposure (less than 2 min) *via* the system presented above. In total, the first stool was analyzed using a direct inoculation of the stool on agar and two enrichments in liquid medium (**Supplementary Table 1**). The second stool was also cultured using a standard culture by direct agar inoculation, as well as with three liquid enrichments using blood culture bottle (**Supplementary Table 1**).

### Culture

#### Standard Culturomics

First, all samples were performed in laminar flow hood at room temperature. The stool samples were aliquoted and frozen at −80°C before use. One gram of stool sample was diluted in 1 ml of phosphate buffered saline (Thermo Fisher Scientific, Illkirch, France) and the diluted samples were introduced with a syringe for pre-incubation into aerobic and anaerobic blood culture bottles (BioMerieux, Marcy l’Etoile, France) different culture conditions have been comprehensively detailed in previous publications (Lagier et al., 2015b). Then, the blood culture vials were incubated at 37°C or 28°C depending on the culture conditions (Lagier et al., 2015b). Inoculation on blood agar of the pre-incubated samples in blood culture bottles was performed for one month on different incubation days; Day 1, Day 3, Day 7, Day 10, Day 15, Day 21, Day 30 (Lagier et al., 2012). To this end, the preincubated stool samples were diluted from 10^−1^ to 10^−10^ in phosphate buffer saline (Thermo Fisher Scientific, Illkirch, France) to dilute the sample and identify the maximum number of bacterial species. Then, 50 µl of diluted sample was deposed and homogenized on blood agar plates (Biomerieux, Marcy l’Etoile, France). Each agar plate was incubated for 24 h at 37°C or 28°C (depending on the culture conditions) for aerobic conditions, or 48 h at 37°C or 28°C for anaerobic conditions, mimicked with Zip bag (Oxoid, Dardilly, France) containing an anaerobic GasPak (Becton Dickinson, Le Pont de Claix, France). Bacterial colonies different in appearance, size, or color were subcultured on blood agar (Biomerieux, Marcy l’Etoile, France) (Lagier et al., 2016; Lagier et al., 2018). These agars were then incubated according to the culture condition previously described. Any isolated colony was applied to mass spectrometry for identification.

#### Fast Culturomics

**Figure 1:**
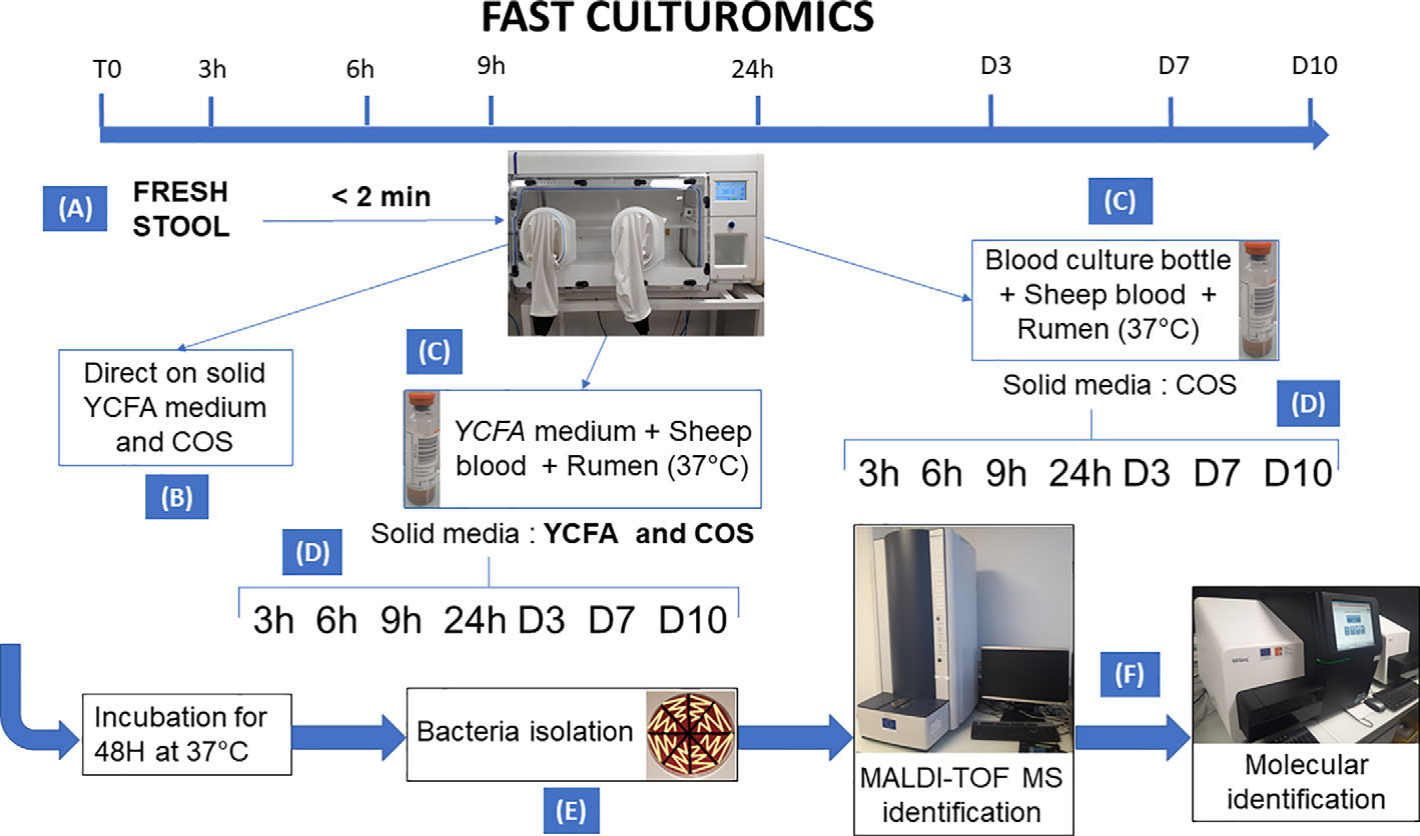
Summary of the methodology of fast culturomics. The fast culturomics was based on six main steps: **(A)** Sampling: Sampling of samples was carried out in anaerobic conditions. For this purpose, the stool sample was placed in a half-open jar, itself placed in anaerobic condition in a zip bag complemented by an anaerobic generator. The assembly was then directly transferred to an anaerobic chamber, after less than 2 min of exposure to oxygen. **(B)** Direct inoculation: direct inoculation of the stool sample was carried out on both blood agar (COS) and YCFA agar. **(C)** Pre-incubation in liquid medium: The sample was pre-incubated in two liquid culture media at 37°C: the commercially available Biomerieux medium and the YCFA medium, both supplemented with 5% sheep blood and 5% rumen filtered at 0.22 µm. **(D)** Inoculation of blood culture bottles on agar: At the different pre-incubation times (3h-6h-9h-24h-D3-D7-D10), a serial dilution of pre-incubated blood culture bottles was performed. Both bottles were inoculated onto blood agar (COS), but only the YCFA+rumen+blood culture condition was also deposited on YCFA agar. **(E)** Subculturing of colonies: Microbial colonies was subcultured after 48 h incubation of the agar at 37°C. This subculturing was carried out according to the different aspects of the microbial colonies obtained. **(F)** Identification: the identification of the microbial species was carried out by Matrix Assisted Laser Desorption Ionization/Time Of Flight Mass Spectrometry (MALDI-TOF) mass spectrometry. If the target species remain unidentified three times, the identification was achieved by molecular biology.

All samples were cultivated in anaerobic chamber at 37°C. A summary of the methodology is shown in **Figure 1**.

##### Direct Inoculation Method

First, a direct inoculation of the stool was performed. To do so, one gram of stool sample was diluted in 1 ml of phosphate buffer saline (Thermo Fisher Scientific, Illkirch, France). Then, 100 μl was taken from the sample, deposited and then homogenized in a second tube containing 900 μl of phosphate buffer saline (Thermo Fisher Scientific, Illkirch, France). This step was carried out several times in order to perform a cascade dilution at 1/10th (from 10^-2^ to 10^-10^). Subsequently, 50 μl of each tube was deposited and spread homogeneously on YCFA modified agar (DSMZ *: Deutsche Sammlung von Mikroorganismen und Zellkulturen*, Germany) (https://www.dsmz.de/microorganisms/medium/pdf/DSMZ_Medium1611.pdf) + 0.5g/L of Na_2_S + 1 g/L of sodium acetate (Martín et al., 2017) + 100 mg/L of Vitamin K2 (Fenn et al., 2017) + 5% of sheep blood (BioMérieux, Marcy l’Etoile, France) and on Columbia agar plates + 5% of blood (Cos, Biomerieux, Marcy l’Etoile, France). The agar plates are then incubated at 37°C under anaerobic condition, mimicked with Zip bag (Oxoid, Dardilly, France) containing an anaerobic GasPak (Becton Dickinson, Le Pont de Claix, France) for 48 h.

##### Patient 1 - Stool Sample 1

A liquid enrichment of the first stool sample at 37°C was performed in two anaerobic blood culture bottles (**Supplementary Table 1**). The first vial contained an anaerobic liquid medium (BioMerieux, Marcy l’Etoile, France) supplemented with 2 ml of rumen fluid filtered at 0.2 μm (Lagier et al., 2015a) and 2 ml of defibrinated and sterile sheep blood (BioMerieux, Marcy l’Etoile, France). The second blood culture bottle contained YCFA modified medium (DSMZ) supplemented with 0.5 g/L Na_2_S, 1 g/L sodium acetate (Martín et al., 2017), 100 mg/L Vitamin K2 (Fenn et al., 2017), 15 mg/L Streptomycin and 20 mg/L of Trimethoprim. Subculture of the initially incubated blood culture bottle was performed after 3 h, 6 h, 9 h, 24 h, 3 days, 7 days, and 10 days of incubation at 37°C. For this, a cascade dilution (from 10^-2^ to 10^-10^) was carried out, 100 μl were taken from the most concentrated preincubated sample, deposited and then homogenized in a second tube containing 900 μl of phosphate-buffered saline (Thermo Fisher Scientific, Illkirch, France). Subsequently, 50 μl of each tube were deposited and spread homogeneously on Columbia agar + 5% blood (Biomerieux, Marcy l’Etoile, France) as well as on YCFA modified agar (DSMZ) + 5% sheep blood (BioMérieux, Marcy l’Etoile, France) for the blood culture bottle containing the modified liquid YCFA medium, and only on blood agar for the blood culture bottle containing the defibrinated and sterile sheep blood (BioMérieux, Marcy l’Etoile, France) and rumen fluid filtered at 0.2 μm (Lagier et al., 2015a). The different plates of agar were then incubated at 37°C under anaerobic conditions for 48 h.

##### Stool Sample 2 Named Megagut

The second stool was analyzed according to the same protocol as the previous one, by direct inoculation and liquid enrichment, except that the blood culture containing the modified YCFA medium (DSMZ) + 0.5 g/L Na2S, 1 g/L of acetate sodium (Martín et al., 2017), 100 mg/L of Vitamin K2 (Fenn et al., 2017), 15 mg/L of Streptomycin and 20 mg/L of Trimethoprim was supplemented with 2 ml of rumen fluid filtered at 0.2μm (Lagier et al., 2015a) and 2 ml of defibrinated and sterile sheep blood (BioMerieux, Marcy l’Etoile, France) (**Supplementary Table 1**). In addition, a third blood culture containing YCFA modified medium (DSMZ) supplemented with 0.5 g/L of Na_2_S, 1 g/L of sodium acetate (Martín et al., 2017), 100 mg/L of vitamin K2 (Fenn et al., 2017) without antibiotics was also tested for this sample and was then inoculated on the two types of agar plates of this work, YCFA (DSMZ) and Columbia agar + 5% blood (BioMerieux, Marcy l’Etoile, France) respectively.

##### Subculturing on Agar Plates

The isolation of the different bacterial colonies in terms of morphological aspect, size or color was carried out and the colonies were deposited on Columbia agar + 5% blood (Biomerieux, Marcy l’Etoile, France) at the rate of 8 colonies per petri dish (Lagier et al., 2012). Then, they were reincubated at 37°C under anaerobic condition.

### Identification

#### Identification by MALDI-TOF Mass Spectrometry

The bacterial identification was performed using the high throughput MALDI-TOF MS technique based on the analysis of ionic entities by their displacement in an electric field (Seng et al., 2009; Seng et al., 2013). The Microflex LT tool (Bruker Daltonik, Bremen, Germany), together with its FlexControl acquisition software and its MALDI Biotyper RTC analysis software, allow to compare the different spectra obtained. For this, we used the Bruker database upgraded by the addition of spectra of previously known bacterial species and new culturomics bacterial species found in our laboratory (Dubourg et al., 2018). These databases contained at the time of submission of this study, 7,854 main spectrum (MSP) (each of main spectrum contained a concatenation of a minimum of 10 spectra) in Bruker database (RUO v.8), supplemented with 6,032 MSP updated in our laboratory (https://www.mediterranee-infection.com/acces-ressources/base-de-donnees/urms-data-base/). If this score was ≥1.9 at the two corresponding spots, the colony was considered identifiable mass spectrometric verification. However, if this score was less than 1.9 or if the results obtained differ for the two bacterial deposits, the identification was considered erroneous. Indeed, a score between 1.7 and 1.9 showed that the identification of the bacterial genus is possible, but the bacterial species identification was not interpretable. In parallel, a score below 1.7 did not allow for possible identification. In this case, the colony is again transplanted and reincubated under appropriate conditions, to be controlled again.

#### Molecular Identification

##### Identification by Sequencing of Ribosomal 16S RNA

The identification of bacterial colonies was initially performed using MALDI-TOF mass spectrometry. However, if identification is not possible and the spectral quality was correct and verified by the Flexanalysis software (Bruker), a Polymerase Chain Reaction targeting the ribosomal 16S RiboNucleic Acid (RNA) was performed, as previously demonstrated (Drancourt et al., 2004; Khamis et al., 2005). A new species was defined by a 16S rRNA sequence homology of less than 98.7% with the closest species proposed by the GenBank (Stackebrandt, 2011).

##### Identification by Genome Sequencing

Genomic DNA was extracted as described above (See *Methods, Section Identification*) and DNA extracts were sequenced using MiSeq technology (Illumina, San Diego, CA, USA) with the paired end strategy and prepared with the Nextera XT Paired end kit (Illumina), as previously described (Lo et al., 2016). The degree of genomic similarity of these two strains with their closely related species was estimated using phylogenetic tree, Digital DNA-DNA hybridization (Auch et al., 2010) and Orthologous Average Nucleotide Identity software (Lee et al., 2016).

## Results

**Figure 2:**
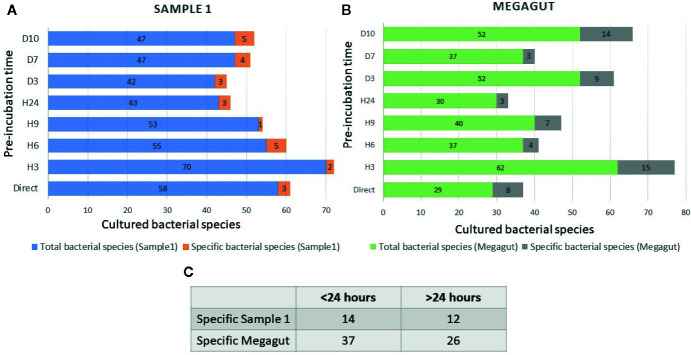
Total and specific number of bacterial species isolated in stool samples as a function of the incubation times. **(A)** Blue histograms represent the total number of species identified in the first stool sample (Sample 1) at different incubation times. The orange histograms show the number of bacterial species only found at different incubation times in the first stool sample (Sample1). **(B)** Green histograms represent the total number of species identified in the second stool sample (Megagut) at different incubation times. The gray histograms show the number of bacterial species only found at different incubation times in the second stool sample (Megagut). **(C)** Table 2C represents the number of bacteria cultured only before or after 24 h of pre-incubation in liquid medium.

Overall, testing 4,988 colonies by MALDI-TOF MS, 121 different bacterial species were cultivated from a standard agar plate culture and two liquid enrichments in stool sample (Sample 1) from a patient requiring rapid immunotherapy treatment (**Supplementary Table 1** and **2**). Of these 121 species, 1 bacterial species was previously unknown from human and 2 bacterial species were unknown to the human gut microbiota (**Supplementary Table 2**). The average number of colonies tested to identify one bacterial species was 41.2 (= 4,988/121), the working time required was 3 weeks, equivalent to 105 working h, for a cost of €2,949. Most of the bacteria isolated were strictly anaerobic bacteria (103/121, 85%) (**Supplementary Table 3**). During this work, 58 bacterial species were cultured using direct plating of the stool on agar and of these 58 bacterial species, 3 bacteria were found only in this culture condition and were lost after pre-incubation in liquid medium (**Figure 2A** and **Supplementary Table 4A**).

**Figure 3:**
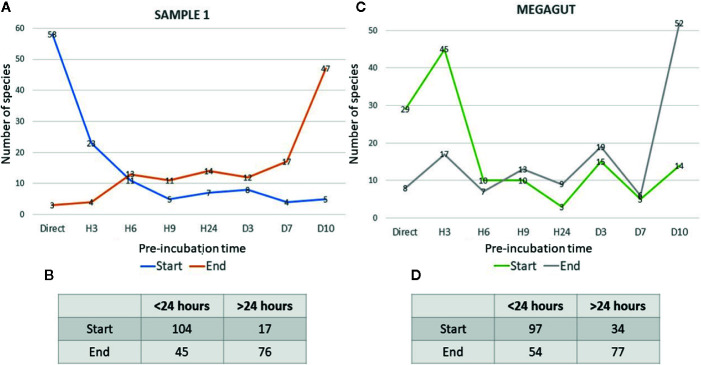
Representative curves of the beginning and the end of the growth of bacterial species identified in stool samples as a function of the incubation time. **(A)** The blue curves represent the number of bacterial species that start growing as a function of the different incubation times in the first stool sample. The orange curves show the number of bacterial species ending their growth as a function of the different incubation times in the first stool sample. **(B)** Table represents the number of bacteria that start or end their growth before or after 24 h in stool sample 1. **(C)** The green curves represent the number of bacterial species that start growing as a function of the different incubation times in Megagut stool sample. The gray curves show the number of bacterial species ending their growth as a function of the different incubation times in the Megagut stool sample. **(D)** Table represents the number of bacteria that start or end their growth before or after 24 h in the second stool sample named Megagut.

In addition, a short pre-incubation in the liquid medium for 3 h, 6 h, 9 h, and 24 h allowed the culture of 70, 55, 53, and 43 different bacterial species, respectively (**Figure 2A** and **Supplementary Table 4A**). A longer pre-incubation of stool in a liquid medium at 37°C for 3, 7, and 10 days induced the culture of 42, 47, and 47 bacterial species (**Figure 2A** and **Supplementary Table 4A**). Therefore, 14/121 bacterial species were identified only before 24 h and were not identified beyond 24 h of pre-incubation in a liquid medium. In parallel, 12/121 bacterial species were identified only after pre-incubation for 24 h (**Figures 2A, C** and **Supplementary Table 4A**). Of the 121 bacterial species cultivated from this sample, 104 started growing within 24 h, while 17 bacterial species started growing after 24 h (**Figures 3A, B** and **Supplementary Table 4A**). In parallel, 45 bacterial species appeared to finish their growth before 24 h against 76 after 24 h (**Figures 3A, B** and **Supplementary Table 4A**). Indeed, **Figure 3** summarizes the duration of growth of the different bacterial species and shows that several bacterial species started and ended their growth before a 24-h pre-incubation. These data demonstrated that not all bacteria require prolonged pre-incubation time to grow, but rather reduced time and could be lost over time.

**Figure 4:**
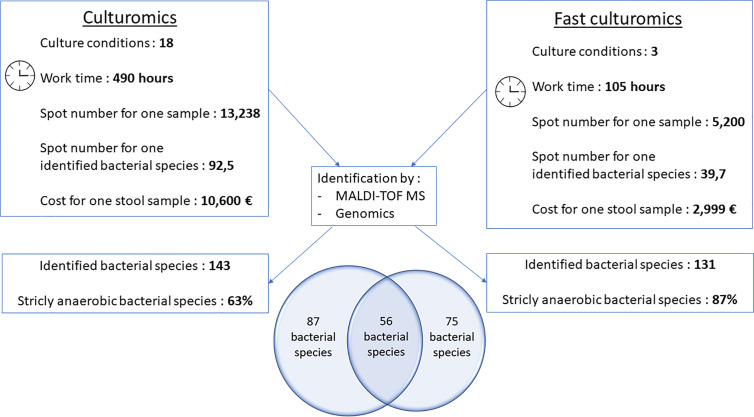
Comparative figure between standard culturomics and rapid culturomics. By adding both standard and fast protocol, 218 bacterial species were identified from the same sample, with 87 bacteria found only when using the 18 culture conditions of the standard culturomics, 75 using only the fast culturomics, and 56 isolated by both techniques.

To confirm the effectiveness of this new method, we conducted the study of the gut microbiota of a second stool sample using this fast culture protocol. It is interesting to note that we were able to compare the results with our basic method of the 18 conditions because we have already tested a sample of the same person (Diakite et al., 2019). During this work, 5,200 colonies were tested by MALDI-TOF MS, allowing the identification of 131 bacterial species (**Figure 4** and **Supplementary Table 2**). An average of 39.7 spots were tested in order to obtain one bacterial species (=5,200/131) (**Figure 4**). This culture was carried out for 3 weeks, representing 105 working h, at a cost of €2,999, and 87% (=114/131) of the cultivated species were strict anaerobic bacterial species (**Figure 4** and **Supplementary Table 3**). Of these 131 species, 3 bacterial species were unknown to the human gut microbiota (**Supplementary Table 2**). Direct inoculation on agar allowed the culture of 29 different bacterial species (**Figure 2B** and **Supplementary Table 4B**). A short pre-incubation in a liquid medium for 3 h, 6 h, 9 h, and 24 h allowed the culture of 62, 37, 40 and 30 different bacterial species, respectively (**Figure 2B** and **Supplementary Table 4B**). A longer pre-incubation for 3, 7 and 10 days allowed the culture of 52, 37 and 52 bacterial species, respectively (**Figure 2B** and **Supplementary Table 4B**). Thus, 37 bacterial species were identified only before 24 h compared to 26 bacterial species after 24 h (**Figures 2B, C** and **Supplementary Table 4B**). Of the 131 bacterial species cultivated, 97 bacterial species started growing before 24 h, while 34 started growing after 24 h (**Figures 3C, D** and **Supplementary Table 4B**). In parallel, 54 bacterial species appeared to have completed their growth before 24 h compared to 77 after 24 h (**Figures 3C, D** and **Supplementary Table 4B**). These results confirm that there are bacterial species that complete their growth at reduced pre-incubation times in liquid medium.

When compared to the gold standard of culturomics, the workload had lasted 14 weeks, representing 490 working h, at a cost of €10,600 (**Figure 4**). We tested 13,238 colonies by MALDI-TOF mass spectrometry and 143 different bacteria were cultured, including 1 new bacterial species named *Lactobacillus caccae* (16S rRNA gene and genome sequences were deposited in Genbank under accession number LT671596.1 and FTOY00000000.1; https://www.ncbi.nlm.nih.gov/) respectively (**Figure 4** and **Supplementary Table 3**). On average, 92.5 colonies tested by MALDI-TOF MS were required to identify one bacterial species (**Figure 4**). Overall, 63% of bacterial species were strict anaerobic (**Figure 4** and **Supplementary Table 3**). When adding both the standard and fast protocol, 218 bacteria were identified from this individual, with 87 bacteria found using only the 18 culture conditions, 75 using only the fast culturomics and 56 isolated by both techniques (**Figure 4**). In conclusion, in order to isolate a comparable number of bacterial species, this new protocol reduces the number of colonies tested by 57%, the work time by 78.6% and the cost for one stool sample by 72.2% (**Figure 4**). The proportion of strict anaerobic species was increased by 24% (**Figure 4**).

## Discussion

Culturomics offers a considerable new perspective on the human gut microbiota knowledge, including recent clinical implications (Lagier et al., 2016; Lagier et al., 2018; Lagier et al., 2019). Nevertheless, so far, the main weaknesses of culturomics are both a significant cost and considerable workload (Lagier et al., 2016). This new culture protocol is faster and cheaper than gold standard culturomics. Using this new fast culturomic protocol, it was possible to cultivate a comparable number of bacterial species in one stool sample. Fast culturomics dramatically reduced the number of colonies tested, the work time and the cost, and increased the proportion of strict anaerobic species. Of these strictly anaerobic bacterial species, this new protocol of culturomics has made it possible to cultivate and identify bacterial species positively associated with the immunotherapy support of a patient treated by anti-PD1 monoclonal antibody (Vétizou et al., 2015; Tanoue et al., 2019). We are confident in our results because we used negative and positive controls, as previously described (Lagier et al., 2018).

First, we compared our data with two significant publications that deal with bacterial species involved in cancer immunotherapy (Vétizou et al., 2015; Tanoue et al., 2019). We showed that with standard culturomics, it was possible to cultivate 2/3 of bacterial species (*Bacteroides thetaiotaomicron* and *Bacteroides uniformis*) associated with anticancer immunotherapy according to Vétizou et al. (2015), and 5/11 bacterial species of anticancer bacteria consortium according to Tanoue et al. (2019) (*Bacteroides uniformis, Parabacteroides distasonis, Parabacteroides johnsonii, Eubacterium limosum, Phascolarctobacterium faecium*). In parallel, with fast culturomics protocol, we cultivated 3/3 (*Bacteroides thetaiotaomicron, Bacteroides fragilis* and *Bacteroides uniformis)* and 6/11 (*Bacteroides uniformis, Parabacteroides distasonis, Parabacteroides gordonii, Alistipes senegalensis, Eubacterium limosum, Phascolarctobacterium faecium*) of bacterial species associated with anticancer immunotherapy (Vétizou et al., 2015; Tanoue et al., 2019). In addition to being faster and cheaper, this technique has made it possible to identify more intestinal bacterial species positively associated with the immunotherapy support by anti-PD1 monoclonal antibody, than standard culturomics.

According to the current scientific literature, it has often been assumed that rare and fastidious bacterial species have a slow growth and can increase their proportion after prolonged incubation time (Rappé et al., 2002; Song et al., 2009, 11). Nevertheless, in this study, the majority of bacteria could be identified within 24 h of their pre-incubation in a liquid medium (**Figures 2** and **3**). Indeed, this new culture technique consists in using the liquid YCFA medium supplemented with defibrinated sheep blood and filtered rumen at 0.22 μm at reduced incubation times in liquid medium (Direct inoculation, 3 h, 6 h and 9 h, 24 h) as well as longer times (3 days, 7 days, and 10 days). We used YCFA medium because previous study showed YCFA medium facilitated the growth of rare and fastidious bacteria (Forster et al., 2019). Other studies have shown that the incubation time does not influence the number of rare bacterial species that can be cultivated (Kato et al., 2018; Kurm et al., 2019). It has also been suggested that several bacterial species may be rare in the community because of a poor competitive ability compared to more abundant species (Hibbing et al., 2010). All of these data suggest a more precise definition of « fastidious bacterial species ».

The strength of this work is that it responds to the disadvantages of standard culturomics. On the one hand, we noticed both a reduction of the workload and working time, as well as the total cost of the experiment. On the other hand, one of the major advantages of this work is that we cultivated fastidious bacterial species involved in human health and diseases. The scientific literature showed that the aforementioned bacterial species required a prolonged pre-incubation time (Holdeman et al., 1976). However, in our study, *Akkermansia muciniphila*, which is involved in the regulation of PD1-based immunotherapy (Routy et al., 2018) and *Barnesiella intestinihominis*, which is involved in cyclophosphamide-induced therapeutic immunomodulatory effects (Daillère et al., 2016) were cultivated in only 3 h in liquid medium (**Supplementary Table 4a** and **Supplementary 4b**). Other target bacteria in biotherapy, such as *Faecalibacterium prausnitzii* (Duncan et al., 2002) and *Alistipes senegalensis* (Tanoue et al., 2019) strains were cultivated after pre-incubation in liquid medium of only 3 h (**Supplementary Table 4a**). This study also allowed the incrementation of our knowledge of the human microbiota as we showed that bacterial species were for the first time cultivated in humans or in the human gut (**Supplementary Table 2**). Nevertheless, this work was a proof of concept and was based only on the analysis of two samples. In addition, it would be useful to consider the use of aerobic culture condition supplemented with, for example, Metronidazole, in order to compensate for the lack of obligate aerobic bacterial species cultured. Moreover, to achieve a complete optimization of this proof of concept, we propose to carry out longer cultures in order to grow bacterial species that require a longer culture time.

In conclusion, for a comparable number of cultivated bacterial species, this new culturomic protocol allows a faster analysis of the intestinal microbiota. It also shows that bacterial species have a precocious grow (a few hours) and disappear over time. In addition, facultative and strict anaerobic bacterial species, that are preferential targets for biotherapy, appear to grow more rapidly and not to be prolonged over time. This is why, in future clinical applications, it would be possible to use this new technique as a pre-therapeutic assessment in patients requiring emergency PD1 treatment in order to select the best protocol for cancer treatment (Routy et al., 2018).

## Data Availability Statement

The datasets presented in this study can be found in online repositories. The names of the repository/repositories and accession number(s) can be found in the article/**Supplementary Material**.

## Ethics Statement

The study was approved by the “Institut Hospitalo-Universitaire Méditerranée Infection”, under agreement number N ° 2016-011. The patients/participants provided their written informed consent to participate in this study.

## Author Contributions

SN performed the experiments and wrote the manuscript. J-CL and DR reviewed the drafts of the manuscript. SK proposed the use of culturomics at reduced times. DM performed the experiments of MALDI-TOF mass spectrometry for rapid culturomics. ND performed the culturomics experiments. J-CL and DR were responsible for the data interpretation and critically revised the manuscript. All authors contributed to the article and approved the submitted version.

## Funding

This study was supported by the National Research Agency under the program « Investissements d’avenir », reference ANR-10-IAHU-03. This work was supported by the Région Provence-Alpes-Côte d’Azur and European funding FEDER PRIMI.

## Conflict of Interest

SK and DR are coinventors of a patent on the culture of anaerobic bacteria (CAS 28-FR1757574). SK, J-CL, and DR are coinventors of a patent for the preservation of bacteria (1H53 316 CAS 25). DR and SK are cofounder of Culture Top.

The remaining authors declare that the research was conducted in the absence of any commercial or financial relationships that could be construed as a potential conflict of interest.
