# Supplementary material for: Proof of Concept of Culturomics Use of Time of Care
Source: Front Cell Infect Microbiol. 2020 Nov 23;10:524769. doi: 10.3389/fcimb.2020.524769 (PMC7719802; doi:10.3389/fcimb.2020.524769)
Supplement: Supplementary file 1 [file Table_1.docx]

**Supplementary Table 1. Representative table of culture conditions used for rapid culture of human stool samples**. Direct inoculation of stool samples was realized in solid YCFA and/or solid blood agar (COS). The inoculation after pre-incubation in liquid medium of stool samples was realized in solid YCFA and/or solid blood agar (COS).

| Sample | Direct inoculation on agar | Pre-incubation in liquid medium | Inoculation after pre-incubation |
| --- | --- | --- | --- |
| Stool 1 | Solid COS | Anaerobic liquid medium (BioMerieux, Marcy l’Etoile, France) + 2 mL of rumen fluid filtered at 0.22 µm + 2 mL of defibrinated and sterile sheep blood | Solid COS |
|  | Solid YCFA | YCFA modified medium + 0.5 g / L of Na_2_S + 1 g / L sodium acetate + 100 mg / L of Vitamin K2 + 15 mg / L of Streptomycin + 20 mg / L of Trimethoprim | Solid COS and solid YCFA |
| Megagut | Solid COS | Anaerobic liquid medium (BioMerieux, Marcy l’Etoile, France) + 2 mL of rumen fluid filtered at 0.22µm + 2 mL of defibrinated and sterile sheep blood | Solid COS |
|  | Solid YCFA | Modified YCFA medium + 0.5 g / L of Na_2_S + 1 g / L of sodium acetate + 100 mg / L of Vitamin K2 + 15 mg / L of Streptomycin + 20 mg / L of Trimethoprim + 2 mL of rumen fluid filtered at 0.22 µm + 2mL of defibrinated and sterile sheep blood | Solid COS and solid YCFA |
|  |  | Modified YCFA medium + 0.5 g / L of Na_2_S + 1 g / L sodium acetate + 100 mg / L of Vitamin K2 | Solid COS and solid YCFA |
